# Increased p‐Tau181 Levels After Overnight Wakefulness Are Associated With Neuroticism in Young Women

**DOI:** 10.1111/jsr.70278

**Published:** 2026-01-23

**Authors:** Diana A. Nôga, Elisa M. S. Meth, Abdullah Almajni, Viviana Rossi, Camilla Zetterlund, Samira F. M. Noory, Michaela Danek, Asma Al‐Grety, André P. Pacheco, Meina Wu, Mia Phillipson, Henrik Zetterberg, Kim Kultima, Pei Xue, Christian Benedict

**Affiliations:** ^1^ Department of Pharmaceutical Biosciences Uppsala University Uppsala Sweden; ^2^ Department of Medical Sciences, Clinical Chemistry Uppsala University, Akademiska Sjukhuset Uppsala Sweden; ^3^ Department of Research and Innovation, Division of Mental Health and Addiction Oslo University Hospital Oslo Norway; ^4^ Institute of Clinical Medicine, Faculty of Medicine University of Oslo Oslo Norway; ^5^ Department of Neurology and Sleep Medical Center Fujian Provincial Governmental Hospital Fuzhou China; ^6^ Department of Medical Cell Biology, the Science for Life Laboratory Uppsala University Uppsala Sweden; ^7^ Department of Psychiatry and Neurochemistry Institute of Neuroscience and Physiology, Sahlgrenska Academy at the University of Gothenburg Mölndal Sweden; ^8^ Clinical Neurochemistry Laboratory Sahlgrenska University Hospital Mölndal Sweden; ^9^ Department of Neurodegenerative Disease UCL Institute of Neurology, Queen Square London UK; ^10^ UK Dementia Research Institute at UCL London UK; ^11^ Hong Kong Center for Neurodegenerative Diseases Hong Kong China; ^12^ Wisconsin Alzheimer's Disease Research Center, School of Medicine and Public Health University of Wisconsin, University of Wisconsin‐Madison Madison Wisconsin USA; ^13^ Centre for Brain Research Indian Institute of Science Bangalore India

**Keywords:** Alzheimer's disease biomarker, estradiol, neuroticism, night shift work, psychomotor vigilance, women

## Abstract

Night shift work can impair attention and increase biomarkers linked to neurodegenerative processes. Understanding factors that influence resilience to, or vulnerability under, sleep loss is therefore essential for identifying groups at particular risk. In this within‐subjects study, we examined two potential modulators of vulnerability in 54 healthy, naturally cycling women aged 21–33 years: the ovarian hormone estradiol, known for its neuroprotective properties and the personality trait neuroticism, previously associated with stress sensitivity. Participants completed one night of habitual sleep followed by one night of overnight wakefulness, mimicking a transition from an off‐ to an on‐night shift. Women with higher morning blood estradiol levels or lower neuroticism (indexed by higher emotional stability scores) exhibited non‐significant tendencies towards faster reaction times during successful psychomotor vigilance test (PVT) trials, which assess sustained attention. Notably, lower neuroticism was also associated with significantly fewer attentional lapses (reaction times ≥ 500 ms) during the PVT, whereas estradiol levels were not. However, neither trait modulated the overall decline in attentional performance observed following the night‐shift condition. In contrast, higher neuroticism—but not estradiol—predicted elevated morning levels of phosphorylated tau at threonine 181 (p‐Tau181), a plasma biomarker associated with Alzheimer's disease–related neurodegenerative processes, after the night shift condition. These findings highlight neuroticism as a psychological factor linked to increased neurobiological sensitivity to overnight wakefulness among women.

## Graphical abstract

Overnight wakefulness, mimicking night shift conditions, impaired morning vigilance in healthy, reproductive‐aged women, regardless of estradiol or neuroticism. However, the overnight wakefulness‐related rise in the Alzheimer's disease blood biomarker p‐Tau181 was greater in women with higher neuroticism, suggesting that neurobiological vulnerability to night shift conditions may vary with personality traits.

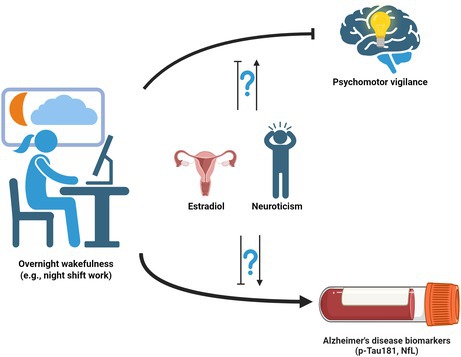

## 1 Introduction

Night shift work is essential in many professions, such as nursing, firefighting and law enforcement. In addition, staying awake overnight is often a necessary part of unpaid caregiving for children or elderly relatives. However, staying awake at night poses multiple health risks for humans (Kecklund and Axelsson 2016), who are naturally diurnal. These risks include, but are not limited to, impaired attention and an increased likelihood of cognitive lapses (Bartel et al. 2004; Maltese et al. 2016; Scott et al. 2006; Vlasak et al. 2022), which may compromise safety and performance in critical occupational settings (Bjerner et al. 1955; Hildebrandt et al. 1974; Smith et al. 1994; Westwell et al. 2021). Moreover, long‐term engagement in night shift work has been linked to an elevated risk of dementia (Jørgensen et al. 2020; Ling et al. 2023). Even under controlled laboratory conditions simulating total sleep deprivation, similar to what night shift workers experience, blood concentrations of biomarkers associated with neurodegenerative diseases such as Alzheimer's disease (AD) (e.g., tau protein; Hampel et al. 2010) have been shown to increase (Benedict et al. 2014, 2020; Lucey et al. 2018; Ooms et al. 2014; Shokri‐Kojori et al. 2018; van Egmond et al. 2022). While these acute elevations may not necessarily indicate permanent neural damage, they highlight the brain's sensitivity to even brief periods of total sleep loss.

Given both the health risks and the high prevalence of night shift work, affecting approximately 13% of the European workforce (IARC Working Group 2020), there is a clear need to identify factors that might modulate vulnerability to its adverse effects on cognitive functioning and brain health. One such factor could be estradiol, a sex hormone predominantly released during the follicular phase and ovulation in women of reproductive age. Evidence from both animal and human studies suggests that estradiol supports cognitive performance and exerts neuroprotective effects, potentially through mechanisms such as reduced neuroinflammation, enhanced synaptic plasticity and mitigated oxidative stress (Albert et al. 2015; Azcoitia et al. 2011; Hara et al. 2015; Luine 2014; Sherwin 2003). Accordingly, it can be hypothesised that women with higher estradiol levels during a night shift may be less susceptible to the attentional impairments and increases in Alzheimer's disease biomarkers typically associated with night work.

Another factor that may influence how overnight wakefulness affects attention and neurodegenerative burden is neuroticism, a personality trait characterised by emotional instability and heightened stress reactivity (Gunthert et al. 1999; Schneider 2004). Since remaining awake during the circadian phase of highest sleep pressure represents a strong physiological and psychological stressor (Minkel et al. 2012, 2014), individuals with higher neuroticism may experience exacerbated cognitive and neurobiological consequences of acute sleep deprivation. Supporting this view, higher levels of neuroticism have been associated with greater mood disturbance, increased sleepiness and poorer attentional performance under conditions of acute sleep deprivation (Duggan et al. 2014; Mastin et al. 2005). Notably, women tend to score higher than men on neuroticism, particularly during young and middle adulthood (Jorm 1987), which may further increase their vulnerability to the cognitive and neurobiological consequences of sleep loss. This sex difference may also partially explain why, despite having objectively better sleep quality than men, women report more frequent symptoms of sleep disturbances (Kocevska et al. 2021).

To test whether staying awake at night affects attention and AD biomarkers in an estradiol‐ and neuroticism‐trait‐dependent manner, in the present study, healthy, reproductive‐aged women participated in a block design across two consecutive days: one night of normal sleep followed by one night of total sleep deprivation, simulating the transition from an off‐night shift to an on‐night shift period. After both nights, attentional capacity was assessed using a psychomotor vigilance task (PVT). In addition, circulating levels of AD biomarkers, phosphorylated tau at threonine 181 (p‐Tau181) and neurofilament light chain (NfL), were measured, as previous studies using total sleep deprivation have shown these parameters may be altered (Benedict et al. 2020; van Egmond et al. 2022).

We hypothesised that women with higher blood estradiol levels at the time of testing would show greater resilience to the experimental night shift condition. Specifically, we expected that they would exhibit fewer attentional impairments and lower blood levels of p‐Tau181 and NfL after sleep deprivation compared to after a night of normal sleep. Conversely, a stronger neuroticism trait, assessed via an emotional stability score in the present study, was hypothesised to be associated with greater attentional deficits and higher AD biomarker levels following overnight wakefulness compared to after sleep.

## 2 Methods

### 2.1 Participants

**FIGURE 1:**
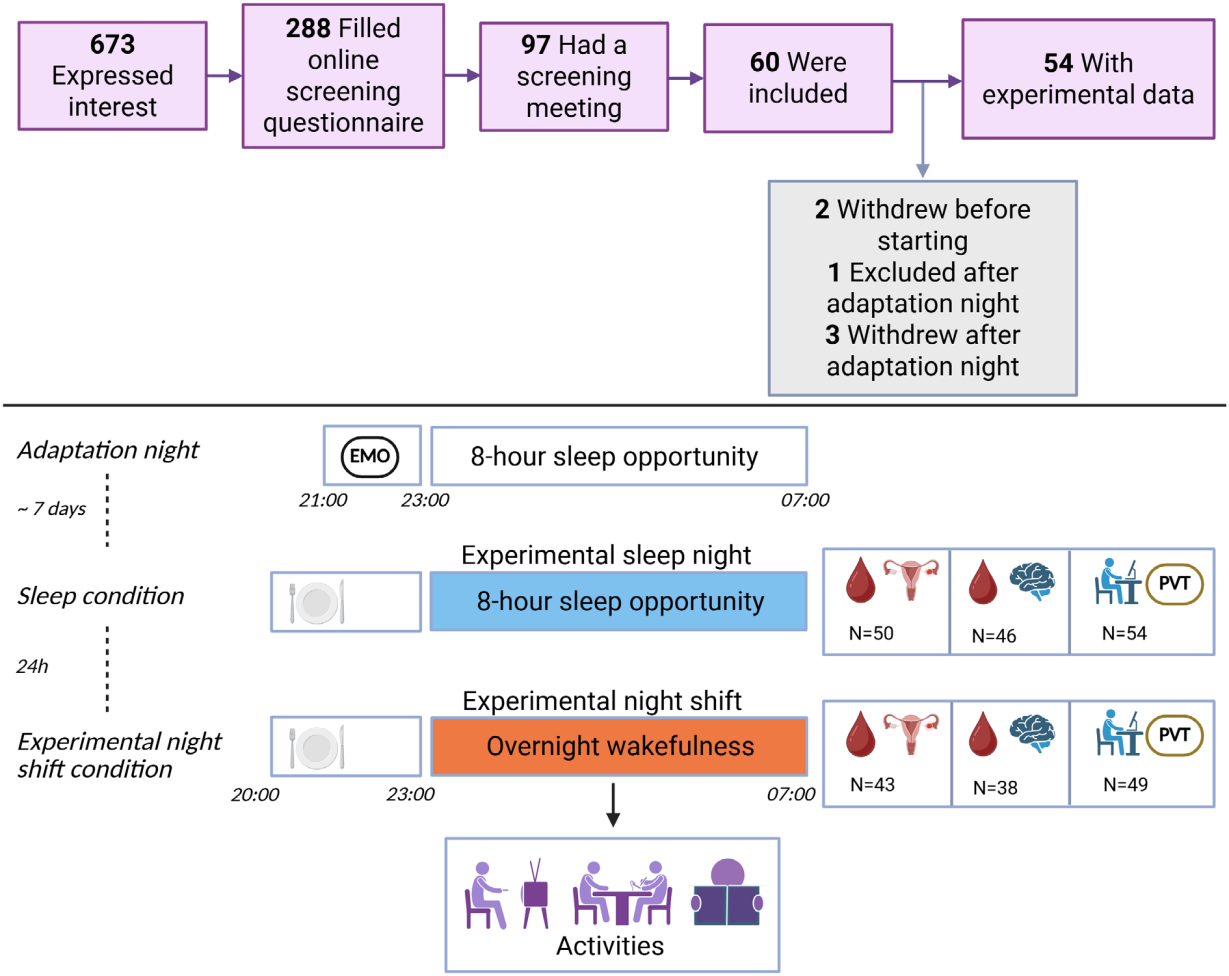
Participant flowchart and experimental design. EMO, emotional stability scale; PVT, psychomotor vigilance task. Blood samples collected at 07:30 for the measurement of female sex hormones (indicated by the blood drop and ovary icons) and Alzheimer's disease biomarkers p‐Tau181 and NfL (indicated by the blood drop and brain icons) were available for only a subset of participants.

As shown in Figure 1, 673 women expressed interest in participating in the study and 288 were subsequently screened for eligibility. Screening involved completing an online questionnaire assessing demographics, health history, sleep habits, menstrual cycle characteristics and lifestyle factors, followed by an in‐person meeting to confirm eligibility and measure participants' height and weight. Exclusion criteria included age below 18 or above 35 years, self‐reported history of physical, psychiatric, or sleep‐related disorders; current or prior (last 6 months) employment involving occasional or permanent night shifts; use of hormonal contraceptives or other chronic medications; irregular menstrual cycles; a menstrual cycle length outside the normal range of 26–35 days; current pregnancy; travel across time zones within 1 month prior to or during the study period; habitual sleep onset before 22:00 or after 00:00; self‐reported bad sleep quality or recurrent insomnia symptoms; habitual sleep duration below 7 or above 9 h; regular nicotine use; and body mass index (BMI) below 18.5 or above 25 kg/m^2^. Details of the online screening questionnaire are provided in the Supporting Information.

Based on eligibility according to our study inclusion criteria, 60 participants were initially included in the study. After two withdrawals, 58 participants underwent an adaptation night in the sleep laboratory. This night was designed to familiarise participants with the laboratory environment and experimental procedures and to reduce potential first‐night effects (Tamaki et al. 2016). Sleep was monitored using a wearable headband electroencephalography (EEG) device (see below). Participants were excluded from the experimental phase if they were unable to sleep during the adaptation night, as determined by either the wearable headband EEG device or self‐report. Additional exclusions were made if an apnea–hypopnea index of five or greater was indicated by the Withings Sleep Analyser, an under‐the‐mattress device used to detect moderate to severe sleep apnea (Edouard et al. 2021). Following these exclusions and participant withdrawals prior to the experimental phase, 54 women (age range: 21–33) participated in at least one of the two experimental sessions.

All study procedures were conducted in accordance with the Declaration of Helsinki and were approved by the Regional Ethical Review Board in Uppsala, Sweden (DNR 66‐2021/3.1). Written informed consent was obtained from all participants prior to enrollment. Participants received monetary compensation for their time and participation. Although the Regional Ethical Review Board did not classify the study as a clinical trial, it was nonetheless registered on ClinicalTrials.gov (NCT06683248) for transparency. Data collection was carried out between May 2022 and July 2024. The analyses presented here represent a subset of a broader investigation into the neurological, metabolic and immune effects of sleep and sleep deprivation.

### 2.2 Experimental Procedure

Approximately 1 week after the adaptation night, participants returned to the laboratory at the Biomedical Centre, Uppsala University, Sweden, for two experimental conditions conducted over two consecutive days. The first condition involved one night of normal sleep (referred to as the sleep condition), while the second involved one night of total sleep deprivation (referred to as the experimental night shift condition). Daily sleep diaries, which tracked bedtimes and wake times during the week leading up to the in‐laboratory sessions, confirmed that participants adhered to their regular sleep–wake schedules.

On the first experimental night (sleep condition), participants arrived at 20:00, received a standardised dinner (~500 kcal), completed a brief set of questionnaires and were fitted with the wearable headband EEG device. Participants then had an approximately 8‐h sleep opportunity (23:00–07:00) in the sleep laboratory. The following morning, they completed the PVT (see below) around 07:45. All 54 participants completed this procedure as described.

The procedure on the second experimental day (experimental night shift condition), which began when participants returned to the laboratory at 20:00, was identical to that of the first day, except that participants were kept awake throughout the night. On the experimental night shift condition, participants engaged in activities such as reading or watching movies, but remained in the laboratory under continuous supervision with ambient illumination maintained at approximately 180 lx at eye level. No food or caffeinated beverages were allowed during the experimental night shift condition. Note that five participants discontinued the study after the first night of sleep or during the experimental night shift condition, in accordance with Swedish ethical regulations, which state that participants may withdraw from an experiment at any time without providing justification. Hence, 49 women completed the experimental night shift condition.

Blood samples for the measurement of estradiol, p‐Tau181 and NfL were collected via venipuncture at 07:30 the morning after each condition. In some cases, venous access was more challenging—for example, due to stress‐related vasoconstriction reducing vein visibility. As a result, the number of available samples varied across assays: estradiol was measured in 50 participants after normal sleep and 43 after sleep loss, while p‐Tau181 and NfL were available from 46 participants after sleep and 38 after sleep loss.

### 2.3 Wearable Headband EEG Device

Sleep was assessed during the adaptation and experimental nights using the Dreem 3 sleep‐monitoring device (Beacon Biosignals Inc., Boston, MA). This wearable, reduced‐montage EEG device features five dry electrodes (O1, O2, FpZ, F7, F8), a three‐dimensional accelerometer and a pulse oximeter. The device automatically scores sleep stages and has been validated against polysomnography, demonstrating high concordance with conventional scoring (Arnal et al. 2020). As shown by Arnal et al. (2020), total sleep time (TST) measured by the Dreem device closely matched polysomnography and overall sleep staging accuracy—including N1, N2, N3, rapid eye movement (REM) and wake after sleep onset (WASO)—reached 83.5% ± 6.4%, compared with an average of 86.4% ± 8.0% for five experienced sleep‐scoring experts.

### 2.4 Female Hormone Assessment

Blood samples for analysis of estradiol (the primary hormone of interest in the present study), progesterone, follicle‐stimulating hormone (FSH) and luteinizing hormone (LH) were collected in SST II tubes (BD, Stockholm, Sweden) and sent to the Clinical Laboratory at Uppsala University Hospital, Sweden. Hormone quantification was performed using a competitive immunoassay on the Roche Cobas Pro analyser (Roche Diagnostics). Participants' menstrual cycle phase at the time of testing was determined based on hormone levels, in accordance with clinical guidelines (Uppsala Clinical Chemistry 2025) and complemented by self‐reported cycle information, including day of last menstruation and average cycle length.

We successfully collected 50 blood samples following the sleep condition and 43 samples following the sleep‐loss condition. Given the absence of significant differences in estradiol levels between conditions (mean ± SD: after sleep, 382.3 ± 268.9 pmol/L; after the sleep loss night, 385.1 ± 238 pmol/L; V = 337.5, *p* = 0.230, Wilcoxon signed‐rank test), we used linear regression to impute missing values for participants with estradiol data from only one condition.

### 2.5 Neuroticism Assessment

Neuroticism was assessed during the adaptation night session using the emotional stability dimension of the Big Five Inventory (John and Srivastava 1999). Lower scores indicate higher levels of neuroticism, whereas higher scores reflect greater emotional stability.

### 2.6 Alzheimer's Disease–Related Biomarkers

Blood samples were collected via venipuncture, using lithium heparin tubes (BD Sweden, Stockholm). Samples were centrifuged at 1300 × g for 10 min at 4°C. The resulting plasma was aliquoted and stored at −80°C until biochemical analysis. The biomarkers p‐Tau181 and NfL were measured using Simoa p‐Tau181 Advantage V2.1 kit and Simoa NF‐light v2 Advantage kit (Quanterix, Billerica, MA, USA), respectively. Blood samples for determining p‐Tau181 and NfL were available from 46 participants after sleep and 38 after sleep loss, with missing samples primarily due to insufficient blood volume or withdrawal of consent. The lower limit of detection (LOD) for NfL was 0.141 ng/L. The LOD for p‐Tau181 was 1.04 ng/L. Values below the LOD were set to the LOD (*n* = 6). All analyses were conducted by the same board‐certified technician at the Department of Clinical Chemistry, Uppsala University Hospital, Sweden.

### 2.7 Psychomotor Vigilance Task

The PVT is a computerised reaction time test widely used to assess objective vigilance and sustained attention (Dinges and Powell 1985). A 3‐min version was employed in this study, previously validated for sensitivity to sleep deprivation (Basner et al. 2011). The task was programmed using the Psychology Experiment Building Language (PEBL; Versions 0.14 and 2.1).

During the task, participants were instructed to fixate on a central cross and press the spacebar on a computer keyboard as quickly as possible in response to a visual stimulus (a red dot), which remained on screen for 400 milliseconds. The stimulus onset varied randomly between 2 and 12 s to prevent anticipatory responses. No performance feedback was provided during the task. Trials with reaction times below 100 milliseconds were classified as false starts and those with reaction times of 500 milliseconds or greater were defined as lapses. Both false starts and lapses were excluded from the calculation of mean reaction time. The primary outcome measures were mean reaction time (ms) and number of lapses.

### 2.8 Statistical Analyses

All statistical analyses were conducted using R (version 4.4.3) or IBM SPSS (version 30). Linear mixed models (LMMs) were employed to examine differences in p‐Tau181, NfL and PVT response time between the experimental night shift and sleep conditions (hereafter referred to as condition). Morning blood estradiol concentrations (blood estradiol) and the emotional stability score (higher scores indicate greater emotional stability; hereafter neuroticism) were included in the models to assess their associations with PVT response time and blood biomarkers. All models included a random intercept for participants.

Initial LMMs tested interactions of interest (condition × blood estradiol and condition × neuroticism) to determine whether the effects of the experimental night shift condition varied by estradiol levels or emotional stability. If interactions were non‐significant, models were simplified to test only main effects.

To assess differences in the number of lapses during the morning PVT session, generalised linear mixed models (GLMMs) with a Poisson distribution and log link were used. Interactions were tested first, with simplified main‐effects models applied if interactions were non‐significant. Finally, sensitivity analyses examined whether TST during the experimental sleep night influenced the above results. For all analyses, two‐tailed *p*‐values < 0.05 were considered statistically significant.

## 3 Results

### 3.1 Cohort Characteristics

As shown in Table 1, our cohort was in their mid‐twenties, had normal weight and university education was the most prevalent. Estradiol levels ranged from 40 to 1177 pmol/L across conditions, progesterone from 0.20 to 67 nmol/L, FSH from 0.86 to 13.10 IU/L and LH from 0.70 to 68 IU/L. Based on these hormonal estimates, the follicular phase was the most common menstrual cycle phase among participants (see Table 1).

**TABLE 1:** Cohort characteristics at the time of the study.

Characteristic	Mean (SD)
Age, years	24.8 (2.6)
BMI, kg/m^2^	22.0 (1.8)
Educational level, *n* (%)	
Non‐university	12 (22.2)
University	42 (77.8)
Menstrual phase, *n* (%)	
Follicular	30 (55.6)
Ovulation	3 (5.6)
Luteal	21 (38.9)

*Note:* Data are shown as mean (SD), unless otherwise specified.

Abbreviation: BMI, body mass index.

During the night prior to the experimental night shift condition, participants were given an 8‐h sleep opportunity in the laboratory. Based on Dreem headband recordings, TST averaged 437 ± 34 min. On average, participants spent 25 ± 11 min in N1, 207 ± 28 min in N2, 98 ± 31 min in N3 and 106 ± 24 min in REM. WASO averaged 22 ± 20 min.

### 3.2 Experimental Night Shift Condition Increased p‐Tau181 in a Neuroticism‐Dependent Manner

**FIGURE 2:**
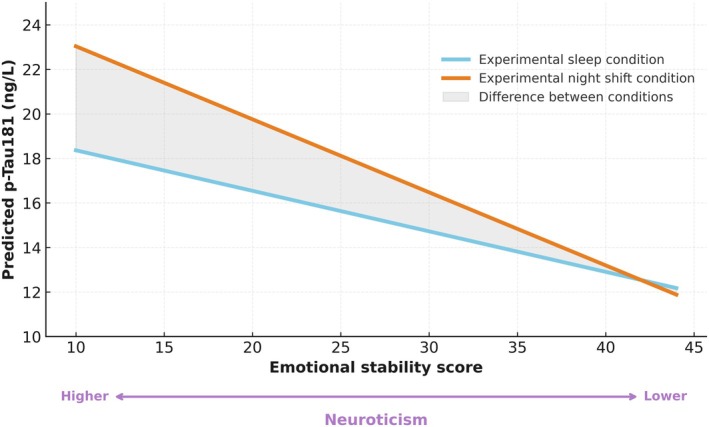
Blood levels of p‐Tau181 after overnight wakefulness (orange) versus after one night of sleep (blue) are higher in reproductive‐aged women with higher neuroticism. Emotional stability scores (higher scores indicate lower neuroticism) plotted against predicted blood p‐Tau181 levels for the experimental sleep (blue) and night shift (orange) conditions. Predicted values were derived from linear mixed‐effects models including the interaction of condition × neuroticism and the main effects of condition, neuroticism, and blood estradiol (set to zero for simplicity). The *x*‐axis represents the observed range of emotional stability scores in our cohort. The shaded area (grey) between the lines represents the estimated difference in p‐Tau181 levels between the experimental conditions across the range of neuroticism scores.

A summary of the LMMs results for the studied AD biomarkers is provided in Table S1. No significant interaction between condition and blood estradiol was observed (*p* = 0.988). In contrast, the condition × neuroticism interaction was significant: higher scores on the emotional stability scale (indicative of lower neuroticism) were associated with a smaller difference in blood p‐Tau181 concentrations between the experimental night shift and sleep conditions (β = −0.146 ng/L, *p* = 0.041; Table S1). Based on estimates from this LMMs, emotional stability scores of 10, 20, 30 and 40 (lower scores indicating higher neuroticism) correspond to estimated p‐Tau181 differences between the experimental night shift and sleep conditions of 4.7, 3.2, 1.8 and 0.3 ng/L, respectively. Predicted differences in p‐Tau181 across the observed range of emotional stability scores are illustrated in Figure 2.

No significant interactions of condition with blood estradiol or neuroticism were found for NfL (*p* ≥ 0.167; Table S1). Subsequent LMMs testing main effects showed that blood concentrations of NfL did not significantly differ between conditions (estimated mean difference: −0.1 ng/L) and were not significantly associated with blood estradiol levels or neuroticism (*p* ≥ 0.203; Table S1).

### 3.3 Experimental Night Shift Condition Reduced Vigilance

No significant interactions between condition × blood estradiol or condition × neuroticism were observed for either reaction time (*p* ≥ 0.330) or number of lapses (*p* ≥ 0.384; Table S1). Consequently, main effects were tested. Reaction time during successful PVT trials was, on average, 36 ms slower after the experimental night shift compared to the sleep condition (*p* < 0.001; Table S1). Mean response time was shorter with higher blood estradiol concentrations (−0.03 ms per additional pmol/L) and with higher emotional stability scores (−1.2 ms per point increase). However, these associations did not reach statistical significance (≥ 0.071; Table S1).

**FIGURE 3:**
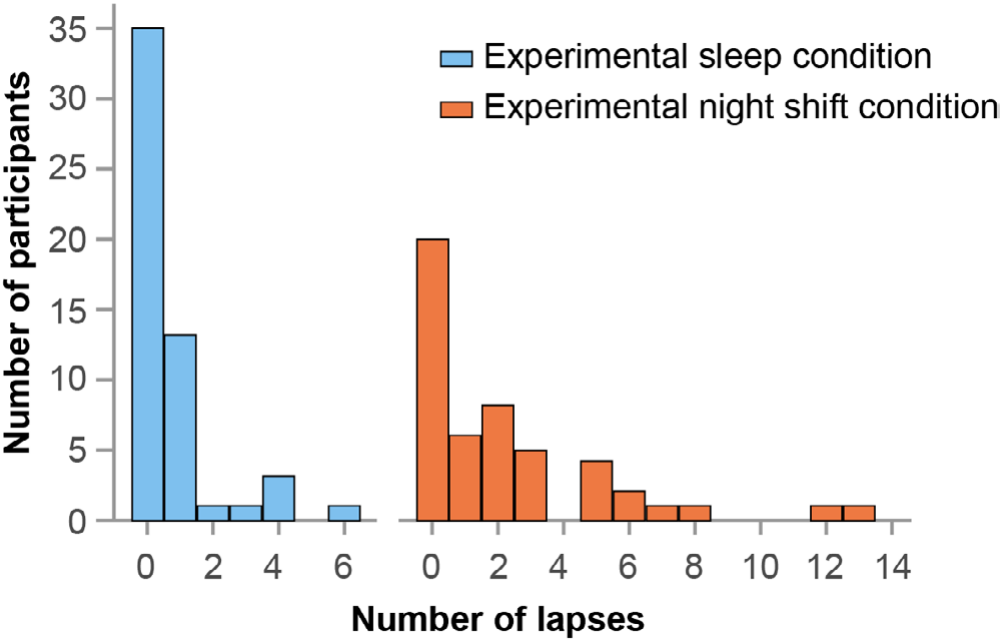
Histogram of total number of lapses during the PVT administered after one night of 8‐h sleep opportunity and one night of total sleep deprivation.

GLMMs revealed no significant interaction effects on the total number of attentional lapses between condition and either blood estradiol or neuroticism (*p* ≥ 0.384). A main effect of condition was observed: participants exhibited, on average, 1.2 lapses more after the experimental night shift compared to the sleep condition (*p* < 0.001; Table S1). Figure 3 shows a histogram of lapses separated by the experimental night shift and sleep conditions. Higher emotional stability scores were associated with fewer lapses (−0.07 per point increase, *p* = 0.002), whereas blood estradiol showed no significant association with lapses (Table S1).

### 3.4 Sensitivity Analyses

When all previous analyses were re‐run including TST from the sleep night as a covariate, the results remained consistent. The significant interaction between emotional stability and condition for p‐Tau181 was maintained (β = −0.15 ng/L, SE = 0.07, *t*(34.57) = −2.14, *p* = 0.039). The main effect of condition on PVT was also robust, as reflected in both reaction time (β = 36.44 ms, SE = 3.7, *t*(46.02) = 9.84, *p* < 0.001) and the number of lapses (β = 1.19, SE = 0.26, *t*(92) = −4.5, *p* < 0.001). Furthermore, a higher emotional stability score remained inversely associated with the number of lapses (β = −0.07, SE = 0.02, *t*(92) = −3.07, *p* = 0.003). No significant main effects of the exposures—including condition, blood estradiol levels, or emotional stability—were observed on NfL, which remained non‐significant (*p* ≥ 0.166) in LMMs testing main effects only.

## 4 Discussion

We found that higher trait neuroticism was significantly associated with increased morning blood levels of p‐Tau181 following overnight wakefulness (the experimental night shift condition) compared with levels observed after a night of in‐laboratory sleep. The mechanisms linking neuroticism to tau phosphorylation are not yet fully understood but may involve physiological stress pathways. Prolonged wakefulness acts as a biological stressor that elevates cortisol, the body's primary glucocorticoid (Leproult et al. 1997). Cortisol, in turn, activates glycogen synthase kinase 3 beta (GSK3β), a key enzyme involved in tau phosphorylation (Lauretti et al. 2020; Yang et al. 2006). Individuals high in neuroticism often exhibit heightened hypothalamic–pituitary–adrenal (HPA) axis reactivity to stress, including exaggerated cortisol responses in standardised laboratory tests (Zobel et al. 2004). Although findings on cortisol reactivity among neurotic individuals are mixed (McCleery and Goodwin 2001; Oswald et al. 2006), this pathway remains a plausible candidate for explaining the current results. An alternative or complementary mechanism may involve inflammation. Neuroticism has been associated with elevated levels of proinflammatory cytokines such as interferon‐gamma (IFN‐γ) (Schmidt et al. 2018), which can promote tau phosphorylation by altering GSK3β regulation (Li et al. 2015). Future studies involving cerebrospinal fluid sampling or broader proteomic and metabolomic analyses are warranted to determine whether cortisol, IFN‐γ, or other central nervous system processes mediate the observed link between neuroticism and increased p‐Tau181 following sleep deprivation.

Given estradiol's well‐established neuroprotective and cognition‐enhancing effects (Albert et al. 2015; Azcoitia et al. 2011; Hara et al. 2015; Luine 2014; Sherwin 2003), we also expected that women with higher blood estradiol levels—measured from morning blood samples—would exhibit smaller increases in AD biomarkers and smaller decreases in attention following the experimental night shift compared with the sleep condition, relative to those with lower estradiol levels. However, no such interaction was observed. This finding may suggest that the adverse effects of night shift work occur relatively uniformly across the menstrual cycle, during which estradiol typically rises in the follicular phase and around ovulation and is lower during the luteal phase and menses (Hawkins and Matzuk 2008). Nevertheless, several factors may have contributed to the null result, including the sample size and the timing of estradiol assessment, which was restricted to morning samples following each experimental night. Future studies should examine how intra‐individual fluctuations in estradiol across the menstrual cycle influence AD biomarker dynamics and attentional performance in the context of experimental night shift paradigms.

Consistent with previous research (Lim and Dinges 2010; Ma et al. 2015), participants demonstrated reduced attentional performance following the experimental night shift condition, reflected in slower reaction times and an increased number of lapses. Women with higher morning blood estradiol levels or lower neuroticism showed non‐significant trends towards faster reaction times on the PVT, a measure of sustained attention (Basner et al. 2011; Dinges and Powell 1985). Importantly, lower neuroticism was significantly associated with fewer attentional lapses (reaction times ≥ 500 ms) during the PVT, whereas estradiol levels were not. However, neither trait moderated the overall decline in attentional performance observed after the experimental night shift condition.

## 5 Limitations

Several limitations should be considered. First, our findings are limited to healthy young naturally cycling women. In designing the study, we sought to simulate the acute effects of a night shift while minimising potential confounding factors—such as caffeine intake, snacking and nocturnal napping—that typically occur during real night shifts and could thereby influence the observed associations. Therefore, our results should not be generalised to individuals experiencing chronic partial sleep loss, such as parents, or those engaged in long‐term shift work, as the study specifically examined acute total sleep deprivation.

Another limitation is that blood samples were unavailable from some participants due to challenges with venous access or withdrawal of consent, which reduced the number of samples available for certain assays. Furthermore, we relied on the Dreem 3 headband's automated sleep staging, which has been validated against polysomnography and provides reliable estimates of TST; however, minor inaccuracies in sleep stage classification have been reported (Arnal et al. 2020). Another consideration is that AD biomarkers were assessed only in the morning, precluding conclusions about whether biomarker changes persist throughout the day or whether overnight fluctuations might have been more pronounced. Finally, although NfL levels did not increase significantly after sleep deprivation—possibly reflecting the biomarker's slower kinetics compared to p‐Tau181 (Khalil et al. 2024)—it is important to note that our study focused specifically on p‐Tau181 and NfL. To gain deeper insight into how night shift conditions may affect brain health, future research should broaden the range of biomarkers assessed, for example by also including AD markers such as amyloid‐beta.

## 6 Conclusions

While attentional deficits resulting from overnight wakefulness do not appear to be moderated by neuroticism or estradiol, women with higher trait neuroticism may still be more vulnerable to the adverse neurobiological effects of overnight wakefulness, as indicated by elevated p‐Tau181, an AD biomarker (Hampel et al. 2010). These findings suggest that night shift–working women with higher neuroticism may benefit from tailored strategies—such as stress‐reduction techniques—and, when feasible, could preferentially avoid night shift work to reduce potential neurological risks.

## Author Contributions

D.A.N., E.M.S.M., V.R., A.P.P., M.P., P.X. and C.B. conceptualised and designed the study. D.A.N., E.M.S.M., A.A., V.R., C.Z., S.F.M.N., M.D., M.W. and P.X. conducted the experiments. D.A.N. coordinated project administration, performed the statistical analyses, interpreted the data and drafted the initial manuscript under the supervision of C.B. D.A.N., P.X., H.Z. and C.B. secured funding for the study. K.K. and A.A.‐G. conducted the laboratory analyses. All authors critically reviewed the manuscript and approved the final version submitted for publication. D.A.N. is the guarantor of this work and, as such, has full access to all study data and takes responsibility for the integrity of the data and the accuracy of the data analysis.

## Funding

This work was supported by the Novo Nordisk Fonden (NNF23OC0081873), the Åke Wiberg Stiftelse (M24‐0251), the Hjärnfonden (FO2023‐0292) and the Vetenskapsrådet (2023‐00356, 2022‐01018, 2019‐02397).

## Conflicts of Interest

H.Z. has served at scientific advisory boards and/or as a consultant for Abbvie, Acumen, Alector, Alzinova, ALZpath, Amylyx, Annexon, Apellis, Artery Therapeutics, AZTherapies, Cognito Therapeutics, CogRx, Denali, Eisai, Enigma, LabCorp, Merck Sharp & Dohme, Merry Life, Nervgen, Novo Nordisk, Optoceutics, Passage Bio, Pinteon Therapeutics, Prothena, Quanterix, Red Abbey Labs, reMYND, Roche, Samumed, ScandiBio Therapeutics AB, Siemens Healthineers, Triplet Therapeutics and Wave; has given lectures sponsored by Alzecure, BioArctic, Biogen, Cellectricon, Fujirebio, LabCorp, Lilly, Novo Nordisk, Oy Medix Biochemica AB, Roche and WebMD; is a co‐founder of Brain Biomarker Solutions in Gothenburg AB (BBS), which is a part of the GU Ventures Incubator Program; and is a shareholder of MicThera (outside submitted work). The remaining authors declare no conflicts of interest related to the content of this article.

## Supporting information


**Data S1:** Supporting Information.
**Table S1:** Summary of key statistics derived from the linear mixed models and the generalised linear mixed models.

## Data Availability

The data is available upon request to researchers from other universities, who are required to sign a data access agreement prior to its release. For inquiries regarding access to the data, please contact diana.noga.morais@uu.se (Diana A. Nôga).
