# Supplementary material for: Increased p‐Tau181 Levels After Overnight Wakefulness Are Associated With Neuroticism in Young Women
Source: J Sleep Res. 2026 Jan 23;35(4):e70278. doi: 10.1111/jsr.70278 (PMC13357770; doi:10.1111/jsr.70278)
Supplement: Supplementary file 1 — Data S1: Supporting Information. Table S1: Summary of key statistics derived from the linear mixed models and the generalised linear mixed models. [file JSR-35-e70278-s001.docx]

**Supplementary Methods**

*Online screening questionnaire*

All participants first completed an online screening questionnaire to assess eligibility for the study. The questionnaire covered the following domains:

- *Demographics*: Age, sex, occupation, and education level.
- *Health history*: Self-reported physical, psychiatric, menstrual cycle-related, and sleep-related disorders, current medications, and hormonal contraceptive use.
- *Sleep habits*: Typical bedtime and wake time, habitual sleep duration, overall sleep quality, and symptoms of insomnia.
- *Menstrual cycle characteristics*: Regularity and typical cycle length, pregnancy.
- *Lifestyle factors*: Nicotine use, caffeine and alcohol consumption, exercise habits, and recent travel across time zones.
- *Night shift history*: Current or prior employment involving night shifts within the last six months.

Responses were reviewed to determine initial eligibility, which was subsequently verified during an in-person screening visit.

**Table S1. Summary of key statistics derived from the linear mixed models and the generalized linear mixed models**

*A) Key statistics derived from the LMMs and GLMMs testing the interaction between condition and blood estradiol.*

| **Outcome, unit** | **Condition * Blood Estradiol** | | **Condition ^#^** | | **Blood Estradiol** | | **Neuroticism ^§^** | |
| --- | --- | --- | --- | --- | --- | --- | --- | --- |
|  | β (SE) | t (df), p-value | β (SE) | t (df), p-value | β (SE) | t (df), p-value | β (SE) | t (df), p-value |
|  |  |  |  |  |  |  |  |  |
| p-Tau181, ng/L**^A^** | −.00 (.002) | -.016 (35.54), .988 | 1.70 (1.04) | 1.64 (34.67), .109 | -.001 (.003) | -.57 (74.98), .568 | -.10 (.13) | -.80 (42.33), .429 |
|  |  |  |  |  |  |  |  |  |
| NfL, ng/L**^A^** | -.001 (.001) | -1.41 (35.04), .167 | .28 (.31) | .91 (34.47), .372 | .000 (.001) | -.52 (67.33), .603 | .06 (.05) | 1.31 (43.18), .199 |
|  |  |  |  |  |  |  |  |  |
| PVT - Reaction time, ms**^A^** | -.02 (.015) | -.99 (45.28), .330 | 42.25 (7.03) | 6.01 (45.51), **<.001** | -.021 (.016) | -1.32 (91.82), .191 | -1.05 (.63) | -1.81 (46.87), .076 |
|  |  |  |  |  |  |  |  |  |
| Lapses, n**^B^** | .001 (.001) | .88 (92), .384 | .83 (.462) | 1.793 (92), .076 | .000 (.001) | -.428 (92), .670 | -.072 (.02) | -3.4 (92), **<.001** |

*B) Key statistics derived from the LMMs and GLMMs testing the interaction between condition and neuroticism.*

| **Outcome, unit** | **Condition * Neuroticism** | | | **Condition ^#^** | | **Blood Estradiol** | | **Neuroticism ^§^** | | |
| --- | --- | --- | --- | --- | --- | --- | --- | --- | --- | --- |
|  | β (SE) | t (df), p-value | β (SE) | | t (df), p-value | β (SE) | t (df), p-value | β (SE) | t (df), p-value |  |
|  |  |  |  | |  |  |  |  |  |  |
| p-Tau181, ng/L**^A^** | -.15 (.07) | -2.12 (34.58), **.041** | 6.13 (2.17) | | 2.83 (34.32), **.008** | -.001 (.0002) | -.29 (75.99), .776 | -.04 (.132) | -.27 (48.03), .791 |  |
|  |  |  |  | |  |  |  |  |  |  |
| NfL, ng/L**^A^** | -.01 (.02) | -0.53 (34.93), .600 | .28 (.72) | | .39 (34.69), .702 | -.001 (.001) | -.82 (72.67), .418 | .07 (.048) | 1.38 (47.68), .174 |  |
|  |  |  |  | |  |  |  |  |  |  |
| PVT - Reaction time, ms**^A^** | -.48 (.52) | -.91 (44.42), .366 | 51.15 (16.61) | | 3.08 (44.09), **.004** | -.025 (.015) | -1.66 (84.28), .101 | -.93 (.677) | -1.38 (62.21), .173 |  |
|  |  |  |  | |  |  |  |  |  |  |
| Lapses, n**^B^** | -.012 (.035) | -.341 (92), .734 | 1.52 (1.02) | | 1.49 (92), .140 | .000 (.001) | .16 (92), .607 | -.06 (.037) | -1.52 (92), .132 |  |

*C) Key statistics derived from the LMMs and GLMMs testing main effects only.*

| **Outcome, unit** | **Condition ^#^** | | **Blood Estradiol** | | **Neuroticism ^§^** | |
| --- | --- | --- | --- | --- | --- | --- |
|  | β (SE) | t (df), p-value | β (SE) | t (df), p-value | β (SE) | t (df), p-value |
|  |  |  |  |  |  |  |
| p-Tau181, ng/L**^A^** | 1.7 (.5) | 3.15 (35.12), **.003** | -.002 (.002) | -.62 (78.53), .538 | -.1 (.1) | -.80 (42.39),.427 |
|  |  |  |  |  |  |  |
| NfL, ng/L**^A^** | -.1 (.2) | -.57 (35.12), .577 | -.001 (.001) | -.95 (72.16), .344 | .06 (.05) | 1.29 (43.14), .203 |
|  |  |  |  |  |  |  |
| PVT - Reaction time, ms**^A^** | 36.0 (3.7) | 9.77 (45.41), **<.001** | -.03 (.01) | -1.77 (85.42), .081 | -1.2 (.6) | 1.85 (47.04), .071 |
|  |  |  |  |  |  |  |
| Lapses, n**^B^** | 1.2 (.3) | 4.44 (93.00), **<.001** | .000 (.001) | .44 (93.00), .659 | -.07 (.02) | -3.26 (93.00), **.002** |

**^A^** Statistical figures derived from linear mixed models (LMMs); **^B^** Statistical figures derived from generalized linear mixed models (GLMMs). **^#^** Experimental night shift vs. experimental sleep condition (set to zero). ^§^ Based on the emotional stability score where higher values correspond to lower neuroticism trait. *Abbreviation*: β, parameter estimate; SE, standard error; t, t-value; df, degrees of freedom; PVT, psychomotor vigilance test; NfL, neurofilament light. P<0.05 is shown in bold.
